# New insights into the immune functions of podocytes: the role of complement

**DOI:** 10.1186/s40348-023-00157-3

**Published:** 2023-04-15

**Authors:** Valentina Bruno, Anne Katrin Mühlig, Jun Oh, Christoph Licht

**Affiliations:** 1 Division of Nephrology, The Hospital for Sick Children, Toronto, ON Canada; 2 Department of Paediatrics, University of Toronto, Toronto, ON Canada; 3 Cell Biology Program, Research Institute, The Hospital for Sick Children, Toronto, ON Canada; 4 University Children’s Research@Kinder-UKE, University Medical Center Hamburg-Eppendorf, Hamburg, Germany; 5 Department of Pediatric Nephrology, University Children’s Hospital, University Medical Center Hamburg-Eppendorf, Hamburg, Germany; 6 III. Department of Medicine, University Medical Center Hamburg-Eppendorf, Hamburg, Germany

**Keywords:** Podocyte, Complement, Immune system

## Abstract

Podocytes are differentiated epithelial cells which play an essential role to ensure a normal function of the glomerular filtration barrier (GFB). In addition to their adhesive properties in maintaining the integrity of the filtration barrier, they have other functions, such as synthesis of components of the glomerular basement membrane (GBM), production of vascular endothelial growth factor (VEGF), release of inflammatory proteins, and expression of complement components. They also participate in the glomerular crosstalk through multiple signalling pathways, including endothelin-1, VEGF, transforming growth factor β (TGFβ), bone morphogenetic protein 7 (BMP-7), latent transforming growth factor β-binding protein 1 (LTBP1), and extracellular vesicles.

Growing literature suggests that podocytes share many properties of innate and adaptive immunity, supporting a multifunctional role ensuring a healthy glomerulus. As consequence, the “immune podocyte” dysfunction is thought to be involved in the pathogenesis of several glomerular diseases, referred to as “podocytopathies.” Multiple factors like mechanical, oxidative, and/or immunologic stressors can induce cell injury. The complement system, as part of both innate and adaptive immunity, can also define podocyte damage by several mechanisms, such as reactive oxygen species (ROS) generation, cytokine production, and endoplasmic reticulum stress, ultimately affecting the integrity of the cytoskeleton, with subsequent podocyte detachment from the GBM and onset of proteinuria.

Interestingly, podocytes are found to be both source and target of complement-mediated injury. Podocytes express complement proteins which contribute to local complement activation. At the same time, they rely on several protective mechanisms to escape this damage. Podocytes express complement factor H (CFH), one of the main regulators of the complement cascade, as well as membrane-bound complement regulators like CD46 or membrane cofactor protein (MCP), CD55 or decay-accelerating factor (DAF), and CD59 or defensin. Further mechanisms, like autophagy or actin-based endocytosis, are also involved to ensure podocyte homeostasis and protection against injury.

This review will provide an overview of the immune functions of podocytes and their response to immune-mediated injury, focusing on the pathogenic link between complement and podocyte damage.

## Background

Podocytes are highly specialized epithelial cells of the glomerulus and represent a major component of the GFB [1]. They have a complex architecture including a large cell body facing the urinary space and an interdigitating network of extensions (primary and secondary processes) terminating as (tertiary) foot processes on the GBM [2].

**Fig. 1:**
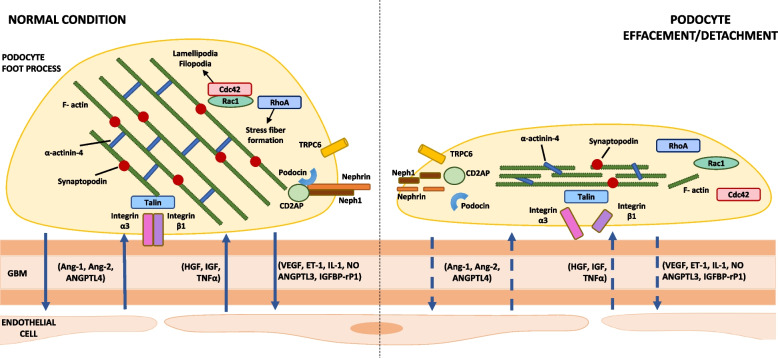
Main components of the slit diaphragm and podocyte-endothelial cell cross talk in healthy versus damaged podocytes. Podocyte slit diaphragm, glomerular basement membrane (GBM), and endothelial cells are the main components of the glomerular filtration barrier. Podocyte effacement/detachment, secondary to mechanical, oxidative, and/or immunologic triggers, is characterized by loss of silt diaphragm integrity, disruption of actin cytoskeleton and focal adhesions, and interruption of the physiological podocyte-endothelial cell cross talk (dashed arrows). Abbreviations: GBM, glomerular basement membrane; Ang, angiopoietin; ANGPTL, angiopoietin-like protein; IGF, insulin-like growth factor; IGFBP-rP1, insulin-like growth factor-binding protein-related protein 1; ET-1, endothelin-1; HGF, hepatocyte growth factor; IL-1, interleukin-1; NO, nitric oxide; TNF-a, tumor necrosis factor-a; VEGF, vascular endothelial growth factor

Normal podocyte function is guaranteed by a sophisticated actin cytoskeleton, mainly localized within the foot processes [3]. Podocytes are characterized by a highly complex architecture regulated by multiple proteins, grouped into two main podocyte structures: the slit diaphragm (SD) and focal adhesions (FA). The SD is a unique highly specialized cell–cell junction between two podocyte foot processes (Fig. 1), including key proteins like nephrin, podocin, or synaptopodin [4, 5]. The SD represents not only a size-selective barrier to prevent filtration of large macromolecules but also a signalling platform with critical functions, such as regulation of the actin cytoskeleton and initiation of signalling pathways to modulate the plasticity of foot processes [6]. FA are complex structures which are able to connect the actin cytoskeleton of foot processes to the GBM, thanks to two main molecular components: integrins and GTPases.

Besides contributing to the GFB, podocytes play important functions such as synthesis and repair of the GBM (together with endothelial cells), production of VEGF, and platelet-derived growth factor (PDGF) [6–9]. Moreover, growing literature suggests that podocytes have many functions of the innate and adaptive immune systems [10–13]. They express cytokine and chemokine receptors to respond to a variety of soluble mediators. They are also able to synthesize inflammatory mediators, such as interleukin-1 (IL-1), which may contribute to local inflammation. Evidence in literature suggests a possible role in the adaptive immune system too, as antigen-presenting cells (APC) to initiate specific T-cell responses, like dendritic cells or macrophages [14, 15].

Furthermore, podocytes express several complement components, such as complement receptor type 1 (CR1) and type 2 (CR2) and complement regulators like CD46, CD55, or CD59, and they can produce complement proteins locally, including complement component 3 (C3) and CFH [16–18]. Nevertheless, the role of complement components expressed or secreted by podocytes in regulation of the local complement reaction is not fully understood.

Podocyte injury is involved in the pathophysiology of several glomerular diseases, like immune-complex glomerulonephritis, minimal change disease (MCD), focal segmental glomerulosclerosis (FSGS), and collapsing glomerulopathy [19, 20], and evidence from the literature suggests that the complement system could be primary or secondary involved in the podocyte damage [21–23].

## The immune podocyte: innate and adaptive functions

Increasing evidence suggests that podocytes play a role in the innate immune response because of their expression of Toll-like receptors (TLRs), especially TLR4, a subtype able to recognize bacterial lipopolysaccharide (LPS). Those receptors are upregulated in animal models of cryoglobulinemic membranoproliferative glomerulonephritis, and they could mediate glomerular damage by modulating expression of chemokines [12].

TLRs are located on the cell surface or intracellularly and can be expressed by different types of cells, such as dendritic cells, macrophages and monocytes, fibroblasts, B and T cells, and endothelial and epithelial cells. They play an essential role by recognizing pathogen-associated molecular patterns; in particular, cell surface TLRs can mainly recognize microbial membrane components such as LPS, lipids, and proteins, while intracellular TLRs mainly recognize nucleic acids from bacteria and viruses [24]. In addition, TLRs can be activated by endogenous ligands released during stress or tissue injury, such as heat shock proteins, mRNA, and necrotic debris [25]. Cultured human podocytes constitutively express cell surface TLRs (i.e., TLR1, 2, 3, 4, 5, 6, and 10) [26], suggesting a possible role in the defense against microbial agents; however, de novo expression of intracellular TLRs subtype has also been reported in podocytes of patients with glomerular disease. In particular, puromycin aminonucleoside (PAN), commonly used to induce a nonimmune podocyte injury in vitro, can upregulate TLR9 intracellular expression and activate NF-κB and p38 MAPK in human immortalized podocytes, utilizing endogenous mtDNA as TLR9 ligand to facilitate podocyte apoptosis [27]. This would suggest a bivalent role of TLRs in podocytes, both as major players in response to foreign pathogens and mediators of podocyte damage.

Moreover, podocytes can express MHC class I and II genes [28, 29], as well as B7-1 (or CD80, involved in T-cell activation) [15, 30] and FcRn (IgG and albumin transport receptor, used by podocytes to internalize IgG from the GBM) [31, 32]. In particular, MHC class II expression on podocytes is required for the development of immune-mediated renal injury, as MHC II presentation by podocytes is necessary to induce the CD4 + T-cell-driven glomerular disease [14]. It is reported that these cells can act as antigen-presenting cells (APC), as they can express several macrophagic-associated markers [33, 34], and they are able to process antigens to initiate specific T-cell responses [15], supporting their multifunctional role in the immunological pathogenesis of glomerular diseases.

Furthermore, expression of functional chemokine receptors (CCR4, CCR8, CCR9, CCR10, CXCR1, CXCR3, CXCR4, and CXCR5) has been demonstrated in cultured human podocytes [35, 36]. Chemokines are small chemoattractant cytokines released by innate immune cells (i.e., neutrophils, eosinophils, macrophages, dendritic cells, natural killer cells), as well as endothelial and epithelial cells. They play a central role in inflammation and immune cell recruitment by guiding circulating leukocytes to inflammation or damage site [37, 38]. They also promote cell growth and tumor angiogenesis and are able to modulate apoptosis by binding G-protein-coupled receptors (GPCRs) on the surface of immune cells. Chemokine receptors are expressed in leukocytes, as well as non-hemopoietic cells, such as endothelial and epithelial cells [39].

CXCR1, CXCR3, and CXCR5 chemokine receptors have been identified in podocytes from kidney biopsies of patients with primary membranous nephropathy (PMN), while they were not expressed in healthy kidneys. Huber et al. suggested that podocyte CXCRs activation may contribute to GFB disruption and onset of proteinuria in PMN through hyperactivation of NADPH oxidases and oxygen radicals production [36].

Podocytes are involved in the inflammatory response of several human glomerulopathies, as suggested by their ability to produce pro-inflammatory cytokines like IL-1α and IL-1β [40, 41]. It has been reported that they can express inflammasome components, like NOD-like receptor (NLR) family proteins, which contribute to inflammatory response in the local kidney in primary glomerular diseases like lupus nephritis (LN) [42].

Podocytes are also known to secrete and/or express several complement proteins and regulators, suggesting local activation of the complement cascade. Expression of complement genes, including C1q, C1r, C2, C3, C3a receptor (C3aR), C5a receptor (C5aR), C7, CR1, and CR2, has been detected in cultured podocytes under normal physiological conditions, with increased local synthesis of complement proteins following podocyte injury [16, 17]. On the other side, complement regulators have been identified too, both membrane-bound (CD46, CD55, CD59) and soluble (CFI and CFH) forms. In particular, podocytes can express CFH locally to clear subendothelial immune complex deposits [43]. The fact that podocytes are able to produce complement components, including regulators, might have a relevant impact on podocytopathies where the complement system plays a pathogenic role. The balance between local complement activation and regulation is important to maintain the glomerular environment, as podocytes could become both target and source of injury, contributing to local complement activation and amplifying their own damage [44, 45].

A summary of the main immune functions of podocytes are summarized in Table 1.

**Table 1:** Summary of (potential and recognized) podocyte immune functions

Molecules	Expression	Immune function and possible implications	References
CD80 (or B7-1)/CD86 Class I/II MHC	Cultured human podocytes can express antigen-presenting cell molecules	Activation of specific T-cell immune responses in renal diseases	[14, 15, 28–30, 33, 34]
Chemokine receptors (CCR and CXCR)	CCR4, CCR8, CCR9, CCR10, CXCR1, CXCR3, CXCR4, and CXCR5 are expressed in cultured human podocytes CXCR1, CXCR3, and CXCR5 have been identified on podocytes from kidney biopsies of PMN patients	Possible pathogenic role in acute and chronic glomerular inflammation NADPH-oxidases hyperactivation and ROS production — possible contribution to glomerular filtration barrier damage, and onset of proteinuria	[12, 35–39]
Complement system components	Cultured human podocytes can produce and express complement components, including regulators	Possible local activation of the complement cascade	[16, 17, 43]
FcRn	Both in vitro and in vivo podocytes express FcRn	IgG clearance from the glomerular basement membrane, albumin recycling	[31, 32]
Cytokines/growth factors/inflammasone components	Both in vitro and in vivo podocytes produce cytokines and growth factors (i.e., TNF-α, IL-1α and β, IL-6, IL-8, VEGF). They can also express inflammasome components (NOD-like receptor family proteins)	Possible role in the local inflammatory response in glomerular diseases	[40–42]
Toll-like receptors (TLRs)	Constitutive expression of cell surface TLRs has been identified on cultured human podocytes De novo expression of intracellular TLRs has been detected in podocytes of patients with glomerular disease (upregulation of intracellular TLR9 with activation of NF-κB/p38 MAPK)	Possible role in the defense against microbial agents Possible role in immune response and glomeruli inflammation	[12, 13, 24–26]

*Abbreviations*: *PMN* primary membranous nephropathy, *FcRn* neonatal Fc receptor, *VEGF* vascular endothelial growth factor, *NOD* nucleotide-binding and oligomerization domain, *NF-κB* nuclear factor-κB, *MAPK* mitogen-activated protein kinase

## Podocyte and complement system

The complement system, classically described as part of the innate immune system, represents indeed a functional bridge between innate and adaptive immunity. It consists of more than 30 plasma or membrane-anchored proteins and regulators which play a role in inflammation, opsonization and lysis of pathogens, clearance of apoptotic cells, and enhancement of both innate and adaptive immunity [46–48]. It can be activated by three different pathways, the classical, the lectin, and the alternative pathway [49, 50], which are tightly regulated by several complement components, like the membrane-bound proteins CD46, CD55, and CD59 and the soluble CFH, to prevent uncontrolled complement hyperactivation [51]. All three pathways induce a proteolytic cascade leading to a shared terminal pathway with subsequent membrane attack complex (MAC) assembly in the cell plasma membrane. Once inserted in the lipid bilayer, MAC forms a stable pore with ~ 10 nm diameter generating several intracellular signals, which have been characterized by both in vivo and in vitro models as summarized in Table 2 [52].

**Table 2:** Signalling pathways activated by MAC (adapted from Takano et al. (2013). Seminars in Nephrology. Reference [52]

Pathway	Effects of terminal pathway activation
Intracellular calcium	Calcium influx through MAC and calcium release from intracellular storage sites
Protein kinases	Activation of protein kinase C (PKC), receptor tyrosine kinase (RTK), Ras-ERK, JNK, p38, and ASK1 (HN)
Phospholipases	Activation of phospholipase C (PLC)-γ1, cPLA2, and iPLA2-γ (phosphorylation), and AA release
Prostanoids	Upregulation of cyclooxygenase (COX)-2 (cultured podocytes and HN glomeruli) and COX-1 (HN glomeruli), production of prostanoids
ROS	Superoxide production via NADPH oxidase and lipid peroxidation (HN) ROS production via xanthine oxidase pathway (HN) Generation of hydrogen peroxide by cytochrome P450 family of hemeprotein monooxygenases (cultured podocytes)
Growth factors	Upregulation of platelet-derived growth factor B-chain, HB-EGF (HN), and Ret (HN and cultured mouse podocytes) Increase of p21 and p27 CDK inhibitors and decrease of CDK2 activity Decrease of p57 and increase of Cdc2, cyclins B1, B2, and D1 and phosphorylated histone-3
Transcription factors DNA damage	Activation of NF-κB (cultured podocytes and in vivo) Production of interleukin-8 and monocyte chemoattractant protein-1 Increase of p21, p53, GADD45, and checkpoint kinase-1 and kinase-2 (cultured podocytes and HN)
Endocytosis Ectocytosis	Endocytosis (podocyte) Ectocytosis in membrane vesicles (urinary space)
ER stress	Damage of ER membrane and unfolded protein response induction Upregulation of ER chaperones, PERK stimulation, eukaryotic translation initiation factor-2α subunit phosphorylation, and reduction of protein synthesis
Ubiquitin–proteasome system	Polyubiquitination of glomerular proteins (HN) Upregulation of ubiquitin proteasome system (cultured podocytes)
Podocyte cytoskeleton	Disassembly of F-actin filaments and focal adhesion complexes Increase of RhoA and decrease of Rac1 and Cdc42 activities (cultured podocytes) Foot process effacement by induction of active RhoA in podocytes (in mice) TRPC6 upregulation (cultured podocytes)
Slit diaphragm	Decrease of nephrin mRNA and protein (HN) Dissociation of nephrin from actin cytoskeleton and loss of slit diaphragm integrity Alteration of podocin location and nephrin dissociation from podocin
Cell cycle	Increased DNA synthesis without cell proliferation (podocyte)
Anti-apoptosis	PI3K/Akt activation, Bad phosphorylation, and dissociation of the Bad/Bcl-XL complex Upregulation of caspase-8 inhibitor and cFLIPL and downregulation of FasL
Pro-apoptosis	DNA damage via apoptosis regulating proteins (podocytes)

*Abbreviations*: *MAC* membrane attack complex, *PKC* protein kinase C, *RTK* receptor tyrosine kinase, *Ras-ERK* Ras-extracellular signal regulated kinase, *JNK* c-Jun N-terminal kinase, *ASK1* apoptosis signal-regulating kinase-1, *HN* Heymann nephritis, *cPLA2* cytosolic phospholipase A2, *iPLA2-γ* independent PLA2-γ, *AA* arachidonic acid, *NADPH* nicotinamide-adenine dinucleotide phosphate, *ROS* reactive oxygen species, *HB-EGF* heparin-binding epidermal growth factor-like factor, *Ret* glial cell-derived neurotrophic factor receptor tyrosine kinase, *CDK* cyclin-dependent kinase, *NF-κB* nuclear factor-κB, *GADD45* growth-arrest DNA damage-45, *ER* endoplasmic reticulum, *PERK* protein kinase R-like ER kinase, *TRPC6* transient receptor potential channel 6, *PI3K* phosphatidylinositol 3-kinase, *Akt* protein kinase B, *Bad* BCL2-associated agonist of cell death, *Bcl-XL* B-cell lymphoma-extra large, *cFLIPL* cellular FLICE-inhibitory protein long form, *FasL* Fas ligand

### Sublytic effects of complement activation on podocytes

Mechanical, oxidative, and immunologic stress can cause podocyte damage and subsequently affect the integrity of glomerular barrier. Complement activation with sublytic MAC formation on podocytes is an example of immunologic stress, which can trigger downstream pathways including protein kinases, lipid metabolism, cytokine production, ROS generation, growth factor signal transduction, endoplasmic reticulum stress, and the ubiquitin–proteasome system, eventually leading to disruption of the podocyte actin cytoskeleton and subsequent cell detachment [53].

More in details, evidence suggests that sublytic amount of MAC on the podocyte surface can induce calcium influx through the membrane pore, as well as calcium release from the intracellular storages, eventually leading to increased intracellular calcium which can activate multiple pathways, such as protein kinase signalling, and in particular protein kinases C (PKC) responsible for membrane vesiculation and internalization of MAC channels [52, 54–57], as suggested by reduction of MAC endocytosis by inhibiting PKC pathway [58].

It is well known that Ca^2+^ signalling in healthy podocytes is mainly mediated by angiotensin II and TRPC5 and 6 (nonselective cationic channels, downstream of angiotensin II signalling) [59]; interestingly, TRPC6 can play a dual role, as it has been shown that acute activation of this channel is able to protect podocytes from complement-mediated injury, while gain-of-function mutations/chronic hyperactivation can affect the SD and/or foot processes morphology leading to glomerular diseases, such as FSGS [60].

It has also been described that sublytic MAC can induce transactivation of receptor tyrosine kinases at the plasma membrane of cultured podocytes, resulting in activation of the Ras-extracellular signal-regulated kinase (ERK) pathway and phospholipase C-γ1. Transactivated receptor tyrosine kinases could play as scaffold for proteins assembly and/or activation, inducing activation of downstream pathways, either dependently or independently the increased cytosolic calcium levels [54, 61].

Other pathways activated by MAC formation on podocyte surface involve arachidonic acid (AA) release by cytosolic phospholipase A2-α (cPLA2), inducing AA metabolism to prostanoids, as described by Cybulsky et al. [62]. Eicosanoids can play a role in complement-mediated podocyte injury, as supported by experimental models of membranous nephropathy. Despite the exact mechanisms of glomerular damage are still unclear, cytotoxic consequences of cPLA2 activation could include release of free fatty acids and lysophospholipids, as well as ions influx, which could ultimately affect the energy machinery [63].

ROS production has also been described in podocytes exposed to sublytic amounts of MAC; both cultured and in vivo podocytes express components of the NADPH oxidase, a complex enzyme able to reduce molecular oxygen to the superoxide anion, which is further metabolized to other ROS [52]. Lipid peroxidation and changes in the podocyte membrane composition, as well as in the GBM components, have been reported as consequence of ROS production. Moreover, inhibition of ROS and/or lipid peroxidation resulted in reduced proteinuria in animal models of membranous nephropathy, suggesting their pathogenic role in glomerular damage [64].

Endoplasmic reticulum (ER) stress with accumulation of misfolded proteins and subsequent increase of the ubiquitin–proteasome system has been reported as additional response to complement-mediated injury, as possible protective response of podocytes to ongoing complement attack [65].

Sublytic MAC deposition on podocytes can also induce DNA damage, both in vitro and in vivo models, as demonstrated by Pippin et al. [66]. The authors also described that sublytic MAC-induced podocyte injury was associated with an increase in specific cell cycle-related genes, including p53, p21, growth-arrest DNA damage-45, and checkpoint kinase-1 and 2, leading to cell cycle arrest and podocyte growth suppression. This could explain why podocyte proliferation is limited following immune-mediated injury.

### Consequences of complement activation on podocyte energy metabolism

The effects of complement activation on podocyte energy machinery are not fully understood. Brinkkoetter et al. demonstrated that podocyte metabolism is somewhat different from other type of cells, as it primarily relies on anaerobic glycolysis and the transformation of glucose to lactate [67]. More in details, the authors showed a significantly lower mitochondrial density per cell area, compared to other type of renal cells (i.e., renal tubular cells). Also, glomeruli stained for mitochondrial enzyme superoxide dismutase 2 (SOD2) and the glycolytic enzyme pyruvate kinase M2 (PKM2) confirmed the perinuclear localization of mitochondria (and their almost complete absence in secondary and tertiary processes), while PKM2 was ubiquitous, suggesting podocyte processes as a large compartment of anaerobic glycolysis. They also used Tfam (mitochondrial transcription factor A) knockout mice to demonstrate that loss of mitochondrial transcription and lack of the oxidative phosphorylation machinery do not induce podocyte disease. In addition, transient knockdown of Tfam in human podocytes significantly reduced mitochondrial respiration, while anaerobic glycolysis was significantly increased allowing a normal podocyte function.

It has been demonstrated that sublytic complement-mediated injury induces reduction of intracellular ATP, in addition to reversible disruption of actin stress fibers and focal adhesions, mainly due to dephosphorylation (instead of degradation) of focal contact proteins, as described by Topham et al. using an in vitro model of rat podocytes [68]; however, the precise mechanisms need to be clarified. Also, complement activation on podocytes can cause nephrin dissociation from the actin cytoskeleton with disruption of the slit diaphragm, GFB damage, and subsequent onset of proteinuria, as suggested by the Heymann nephritis (HN) model [54, 61].

### Complement-mediated injury and podocyte response

Podocytes rely on several adaptive mechanisms to mitigate complement-mediated injury. Autophagy, a highly conserved mechanism of lysosome-mediated degradation of damaged organelles or nonfunctional proteins, is enhanced after sublytic complement damage in mouse podocytes, whereas its inhibition amplifies complement-mediated cell injury [69]. Liu et al. investigated the role of autophagy in PMN, comparing podocytes from PMN patients to cultured mouse podocytes exposed to sublytic complement activity, and they found impaired autophagy in podocytes from PMN patients, characterized by intracellular accumulation of p62 (marker of impaired autophagy) and increase in autophagic vacuoles [70].

Podocyte-derived VEGF has also a bivalent function, as it is described that its overexpression can cause a collapsing glomerulopathy, while its inhibition is associated with GFB disruption, proteinuria, and possible development of thrombotic microangiopathy as well [71]. The putative mechanism is that, in normal conditions, VEGF signalling can regulate complement activity on podocytes and protect them from complement-mediated injury by increasing local CFH production, while its inhibition would provoke reduced levels of CFH, and podocytes would become more vulnerable to the injury.

More recently, new interesting mechanisms have been described to protect podocytes from injury, as reported by Medica et al. using a co-culture model of glomerular endothelial cells and podocytes. In particular, they demonstrated that extracellular vesicles derived from endothelial progenitor cells and involved in intercellular crosstalk (by transferring of proteins, lipids, and genetic material) are able to protect both glomerular endothelial cells and podocytes from complement (C5a)- and cytokine-mediated injury [72]. In particular, they showed that pre-stimulation of endothelial cells with extracellular vesicles prevented podocyte apoptosis and GFB disruption, and this protective effect could be mainly secondary to RNA transfer from the extracellular vesicles to damaged endothelial cells and podocytes.

Despite a tight surveillance of the complement system, including the activity of soluble and membrane-bound regulators, together with the protective mechanisms previously described to escape the injury, unrestricted complement activation can exceed those regulatory mechanisms, causing host tissue injury, as reported in various diseases including glomerulonephritis [73], hemolytic uremic syndrome (HUS) [74], sepsis [75], systemic lupus erythematosus [76], rheumatoid arthritis [77], organ transplant rejection [78], and age-related macular degeneration [79].

## Summary and conclusions

Podocytes play a critical role to ensure the glomerular homeostasis. Over the years, growing literature highlighted the multiple and complex biological functions of these pericytes-like epithelial cells, which are much more than a supporting component of the GFB [1, 80–82].

Several authors described them as “immune podocytes,” to underline their properties as both innate and adaptive immune cells [10, 13, 15]. Understanding their complex biology is essential to unravel the pathogenic mechanisms of several glomerular diseases, where podocyte injury represents a common denominator.

The role of the complement system in podocyte injury has also been evaluated in a multitude of kidney disorders, such as membranous nephropathy, lupus nephritis, HUS, FSGS, and several more [45, 83–90]. The effects of complement activation on podocytes can vary based on the disease pathophysiology, as well as based on the initial trigger, which could induce lytic versus sub-lytic effects. Interestingly, podocytes have developed several protective mechanisms to escape the complement attack, such as autophagy, internalization mechanisms like endocytosis, and expression of complement regulators, and the balance between injury and defense mechanisms can ultimately determine the destiny of the podocyte cell [65, 69, 91].

Future studies, both in vitro and in vivo, are needed to better understand the role of complement activation in podocytopathies and the rationale for the use of anti-complement therapies in conditions where the complement system appears as main driver of the disease.

## Data Availability

Not applicable.

## Abbreviations

APC: Antigen-presenting cells
ATP: Adenosine triphosphate
BMP7: Bone morphogenetic protein 7
C1q: Complement component 1q
C1r: Complement component 1r
C1s: Complement component 1 s
C2: Complement component 2
C3: Complement component 3
C3a: Complement component 3a
C3aR: Complement component 3a receptor
C4: Complement component 4
C5a: Complement component 5a
C5aR: Complement component 5a receptor
C6: Complement component 6
C7: Complement component 7
CCR4: CC chemokine receptor 4
CCR8: CC chemokine receptor 8
CCR9: CC chemokine receptor 9
CCR10: CC chemokine receptor 10
CD80: Cluster of differentiation 80
CFH: Complement factor H
CFI: Complement factor I
cPLA2: Cytosolic phospholipase A2
CR1: Complement receptor type 1
CR2: Complement receptor type 2
CXCR1: CXC chemokine receptor 1
CXCR3: CXC chemokine receptor 3
CXCR4: CXC chemokine receptor 4
CXCR5: CXC chemokine receptor 5
DAF: Decay-accelerating factor
ERK: Ras-extracellular signal-regulated kinase
FA: Focal adhesions
FcRn: Neonatal Fc receptor
FSGS: Focal segmental glomerular sclerosis
GBM: Glomerular basement membrane
GFB: Glomerular filtration barrier
GPCR: G-protein-coupled receptor
HN: Heymann nephritis
HUS: Hemolytic uremic syndrome
IL-1α: Interleukin-1α
IL-1β: Interleukin-1β
LN: Lupus nephritis
LTBP1: Latent transforming growth factor β-binding protein 1
MAC: Membrane attack complex
MAPK: Mitogen-activated protein kinase
MCD: Minimal change disease
MCP: Membrane cofactor protein
MHC: Major histocompatibility complex
NADPH: Nicotinamide adenine dinucleotide phosphate
NF-κB: Nuclear factor kappa-light-chain-enhancer of activated B cells
NLR: NOD-like receptor
PAN: Puromycin aminonucleoside
PDGF: Platelet-derived growth factor
PKC: Protein kinase C
PMN: Primary membranous nephropathy
PKM2: Pyruvate kinase M2
ROS: Reactive oxygen species
SD: Slit diaphragm
SOD2: Superoxide dismutase 2
Tfam: Mitochondrial transcription factor A
TGFβ: Transforming growth factor β
TLRs: Toll-like receptors
TRPC5: Transient receptor potential canonical 5
TRPC6: Transient receptor potential canonical 6
VEGF: Vascular endothelial growth factor

## References

[1] Garg P (2018). A review of podocyte biology. Am J Nephrol.

[2] Miner JH (2011). Glomerular basement membrane composition and the filtration barrier. Pediatr Nephrol.

[3] Schell C, Huber TB (2017). The evolving complexity of the podocyte cytoskeleton. J Am Soc Nephrol.

[4] Kawachi H, Fukusumi Y (2020). New insight into podocyte slit diaphragm, a therapeutic target of proteinuria. Clin Exp Nephrol.

[5] Blaine J, Dylewski J (2020). Regulation of the actin cytoskeleton in podocytes. Cells.

[6] Grahammer F, Schell C, Huber TB (2013). The podocyte slit diaphragm—from a thin grey line to a complex signalling hub. Nat Rev Nephrol.

[7] St. John PL, Abrahamson DR (2001). Glomerular endothelial cells and podocytes jointly synthesize laminin-1 and -11 chains. Kidney International..

[8] Greka A, Mundel P (2012). Cell biology and pathology of podocytes. Annu Rev Physiol.

[9] Byron A, Randles MJ, Humphries JD, Mironov A, Hamidi H, Harris S (2014). Glomerular cell cross-talk influences composition and assembly of extracellular matrix. J Am Soc Nephrol.

[10] Bhargava R, Tsokos GC (2019). The immune podocyte. Curr Opin Rheumatol.

[11] Mathieson PW (2003). What has the immune system got against the glomerular podocyte?. Clin Exp Immunol.

[12] Banas MC, Banas B, Hudkins KL, Wietecha TA, Iyoda M, Bock E (2008). TLR4 links podocytes with the innate immune system to mediate glomerular injury. J Am Soc Nephrol.

[13] Xia H, Bao W, Shi S (2017). Innate immune activity in glomerular podocytes. Front Immunol.

[14] Goldwich A, Burkard M, Ölke M, Daniel C, Amann K, Hugo C (2013). Podocytes are nonhematopoietic professional antigen-presenting cells. J Am Soc Nephrol.

[15] Li S, Liu Y, He Y, Rong W, Zhang M, Li L (2020). Podocytes present antigen to activate specific T cell immune responses in inflammatory renal disease. J Pathol.

[16] Li X, Ding F, Zhang X, Li B, Ding J (2016). The expression profile of complement components in podocytes. Int J Mol Sci.

[17] Mühlig AK, Keir LS, Abt JC, Heidelbach HS, Horton R, Welsh GI, et al (2020) Podocytes produce and secrete functional complement C3 and complement factor H. Front Immunol 11:1833 10.3389/fimmu.2020.01833 PMC7457071 32922395

[18] Angeletti A, Cantarelli C, Petrosyan A, Andrighetto S, Budge K, D'Agati VD, et al (2020) Loss of decay-accelerating factor triggers podocyte injury and glomerulosclerosis. J Exp Med 217(9):e20191699 10.1084/jem.20191699 PMC7478737 32717081

[19] Kopp JB, Anders H-J, Susztak K, Podestà MA, Remuzzi G, Hildebrandt F, et al (2020) Podocytopathies. Nat Rev Dis Prim 6(1):68 10.1038/s41572-020-0196-7 PMC8162925 32792490

[20] Wiggins R-C (2007). The spectrum of podocytopathies: a unifying view of glomerular diseases. Kidney Int.

[21] Huang J, Cui Z, Gu Q-H, Zhang Y-M, Qu Z, Wang X (2020). Complement activation profile of patients with primary focal segmental glomerulosclerosis. PLoS ONE.

[22] Couser WG (2012). Basic and translational concepts of immune-mediated glomerular diseases. J Am Soc Nephrol.

[23] Maillard N, Wyatt RJ, Julian BA, Kiryluk K, Gharavi A, Fremeaux-Bacchi V (2015). Current understanding of the role of complement in IgA nephropathy. J Am Soc Nephrol.

[24] Kawasaki T, Kawai T (2014) Toll-like receptor signaling pathways. Front Immunol 5:461 10.3389/fimmu.2014.00461 PMC4174766 25309543

[25] Karikó K, Ni H, Capodici J, Lamphier M, Weissman D (2004). mRNA is an endogenous ligand for toll-like receptor 3. J Biol Chem.

[26] Srivastava T, Sharma M, Yew K-H, Sharma R, Duncan RS, Saleem MA (2013). LPS and PAN-induced podocyte injury in an in vitro model of minimal change disease: changes in TLR profile. J Cell Commun Signal.

[27] Bao W, Xia H, Liang Y, Ye Y, Lu Y, Xu X (2016). Toll-like receptor 9 can be activated by endogenous mitochondrial DNA to induce podocyte apoptosis. Sci Rep.

[28] Coers W, Brouwer L, Vos JTWM, Chand A, Huitema S, Heeringa P (2008). Podocyte expression of MHC class I and II and intercellular adhesion molecule-1 (ICAM-1) in experimental pauci-immune crescentic glomerulonephritis. Clin Exp Immunol.

[29] Baudeau C, Delarue F, Hé CJ, Nguyen G, Adida C, Peraldi MN (1994). Induction of MHC class II molecules HLA-DR, -DP and -DQ and ICAM 1 in human podocytes by gamma-interferon. Exp Nephrol.

[30] Reiser J, Von Gersdorff G, Loos M, Oh J, Asanuma K, Giardino L (2004). Induction of B7–1 in podocytes is associated with nephrotic syndrome. J Clin Investig.

[31] Akilesh S, Huber TB, Wu H, Wang G, Hartleben B, Kopp JB (2008). Podocytes use FcRn to clear IgG from the glomerular basement membrane. Proc Natl Acad Sci.

[32] Dylewski J, Dobrinskikh E, Lewis L, Tonsawan P, Miyazaki M, Jat PS (2019). Differential trafficking of albumin and IgG facilitated by the neonatal Fc receptor in podocytes in vitro and in vivo. PLoS ONE.

[33] Bariéty J, Nochy D, Mandet C, Jacquot C, Glotz D, Meyrier A (1998). Podocytes undergo phenotypic changes and express macrophagic-associated markers in idiopathic collapsing glomerulopathy. Kidney Int.

[34] Mendrick DL, Kelly DM, Rennke HG (1991). Antigen processing and presentation by glomerular visceral epithelium in vitro. Kidney Int.

[35] Burt D, Salvidio G, Tarabra E, Barutta F, Pinach S, Dentelli P (2007). The monocyte chemoattractant protein-1/cognate CC chemokine receptor 2 system affects cell motility in cultured human podocytes. Am J Pathol.

[36] Huber TB, Reinhardt HC, Exner M, Burger JA, Kerjaschki D, Saleem MA (2002). Expression of functional CCR and CXCR chemokine receptors in podocytes. J Immunol.

[37] Charo IF, Ransohoff RM (2006). The many roles of chemokines and chemokine receptors in inflammation. N Engl J Med.

[38] Proudfoot AEI (2002). Chemokine receptors: multifaceted therapeutic targets. Nat Rev Immunol.

[39] Griffith JW, Sokol CL, Luster AD (2014). Chemokines and chemokine receptors: positioning cells for host defense and immunity. Annu Rev Immunol.

[40] Niemir ZI, Stein H, Dworacki G, Mundel P, Koehl N, Koch B (1997). Podocytes are the major source of IL-1 alpha and IL-1 beta in human glomerulonephritides. Kidney Int.

[41] Wright RD, Beresford MW (2018). Podocytes contribute, and respond, to the inflammatory environment in lupus nephritis. American Journal of Physiology-Renal Physiology.

[42] Xiong W, Meng X-F, Zhang C (2020). Inflammasome activation in podocytes: a new mechanism of glomerular diseases. Inflamm Res.

[43] Zoshima T, Hara S, Yamagishi M, Pastan I, Matsusaka T, Kawano M, et al (2019) Possible role of complement factor H in podocytes in clearing glomerular subendothelial immune complex deposits. Sci Rep 9(1):7857 10.1038/s41598-019-44380-3 PMC6536504 31133737

[44] Tipping PG (2008). Are podocytes passive or provocative in proteinuric glomerular pathology?. J Am Soc Nephrol.

[45] Luo W, Olaru F, Miner JH, Beck LH, van der Vlag J, Thurman JM (2018). Alternative pathway is essential for glomerular complement activation and proteinuria in a mouse model of membranous nephropathy. Front Immunol.

[46] Dunkelberger JR, Song W-C (2010). Complement and its role in innate and adaptive immune responses. Cell Res.

[47] Mathern DR, Heeger PS (2015). Molecules great and small: the complement system. Clin J Am Soc Nephrol.

[48] Ricklin D, Hajishengallis G, Yang K, Lambris JD (2010). Complement: a key system for immune surveillance and homeostasis. Nat Immunol.

[49] Noris M, Remuzzi G (2013). Overview of complement activation and regulation. Semin Nephrol.

[50] Reis ES, Mastellos DC, Hajishengallis G, Lambris JD (2019). New insights into the immune functions of complement. Nat Rev Immunol.

[51] Zipfel PF, Skerka C (2009). Complement regulators and inhibitory proteins. Nat Rev Immunol.

[52] Takano T, Elimam H, Cybulsky AV (2013). Complement-mediated cellular injury. Semin Nephrol.

[53] Nagata M (2016). Podocyte injury and its consequences. Kidney Int.

[54] Cybulsky AV, Quigg RJ, Salant DJ (2005). Experimental membranous nephropathy redux. Am J Physiol Renal Physiol.

[55] Tegla CA, Cudrici C, Patel S, Trippe R, Rus V, Niculescu F (2011). Membrane attack by complement: the assembly and biology of terminal complement complexes. Immunol Res.

[56] Greka A, Mundel P (2011). Balancing calcium signals through TRPC5 and TRPC6 in podocytes. J Am Soc Nephrol.

[57] Cybulsky AV, Bonventre JV, Quigg RJ, Lieberthal W, Salant DJ (1990). Cytosolic calcium and protein kinase C reduce complement-mediated glomerular epithelial injury. Kidney Int.

[58] Carney DF, Lang TJ, Shin ML (1990). Multiple signal messengers generated by terminal complement complexes and their role in terminal complement complex elimination. J Immunol.

[59] Zhang L, Ji T, Wang Q, Meng K, Zhang R, Yang H (2017). Calcium-sensing receptor stimulation in cultured glomerular podocytes induces TRPC6-dependent calcium entry and RhoA activation. Cell Physiol Biochem.

[60] Kistler AD, Singh G, Altintas MM, Yu H, Fernandez IC, Gu C (2013). Transient receptor potential channel 6 (TRPC6) protects podocytes during complement-mediated glomerular disease. J Biol Chem.

[61] Cybulsky AV (2011). Membranous nephropathy. Contrib Nephrol.

[62] Cybulsky AV, Takano T, Papillon J, Mctavish AJ (2000). Complement-induced phospholipase A2 activation in experimental membranous nephropathy1 See Editorial by Shankland, p. 1204. Kidney Int..

[63] Bonventre JV (1992). Phospholipase A2 and signal transduction. J Am Soc Nephrol.

[64] Neale TJ, Ojha PP, Exner M, Poczewski H, Rüger B, Witztum JL (1994). Proteinuria in passive Heymann nephritis is associated with lipid peroxidation and formation of adducts on type IV collagen. J Clin Investig.

[65] Cybulsky AV (2013). The intersecting roles of endoplasmic reticulum stress, ubiquitin–proteasome system, and autophagy in the pathogenesis of proteinuric kidney disease. Kidney Int.

[66] Pippin JW, Durvasula R, Petermann A, Hiromura K, Couser WG, Shankland SJ (2003). DNA damage is a novel response to sublytic complement C5b–9–induced injury in podocytes. J Clin Invest..

[67] Brinkkoetter PT, Bork T, Salou S, Liang W, Mizi A, Özel C (2019). Anaerobic glycolysis maintains the glomerular filtration barrier independent of mitochondrial metabolism and dynamics. Cell Rep.

[68] Topham PS, Haydar SA, Kuphal R, Lightfoot JD, Salant DJ (1999). Complement-mediated injury reversibly disrupts glomerular epithelial cell actin microfilaments and focal adhesions. Kidney Int.

[69] Lv Q, Yang F, Chen K, Zhang Y (2016). Autophagy protects podocytes from sublytic complement induced injury. Exp Cell Res.

[70] Liu WJ, Li Z-H, Chen X-C, Zhao X-L, Zhong Z, Yang C, et al (2017) Blockage of the lysosome-dependent autophagic pathway contributes to complement membrane attack complex-induced podocyte injury in idiopathic membranous nephropathy. Sci Rep 7(1):8643 10.1038/s41598-017-07889-z PMC5561110 28819100

[71] Keir LS, Firth R, Aponik L, Feitelberg D, Sakimoto S, Aguilar E (2016). VEGF regulates local inhibitory complement proteins in the eye and kidney. J Clin Investig.

[72] Medica D, Franzin R, Stasi A, Castellano G, Migliori M, Panichi V (2021). Extracellular vesicles derived from endothelial progenitor cells protect human glomerular endothelial cells and podocytes from complement- and cytokine-mediated injury. Cells.

[73] Kaartinen K, Safa A, Kotha S, Ratti G, Meri S (2019). Complement dysregulation in glomerulonephritis. Semin Immunol.

[74] Noris M, Mescia F, Remuzzi G (2012). STEC-HUS, atypical HUS and TTP are all diseases of complement activation. Nat Rev Nephrol.

[75] Lupu F, Keshari RS, Lambris JD, Mark CK (2014). Crosstalk between the coagulation and complement systems in sepsis. Thromb Res.

[76] Birmingham DJ, Hebert LA (2015). The complement system in lupus nephritis. Semin Nephrol.

[77] Holers VM, Banda NK (2018) Complement in the initiation and evolution of rheumatoid arthritis. Front Immunol 9:1057 10.3389/fimmu.2018.01057 PMC5985368 29892280

[78] Stites E, Le Quintrec M, Thurman JM (2015). The complement system and antibody-mediated transplant rejection. J Immunol.

[79] McHarg S, Clark SJ, Day AJ, Bishop PN (2015). Age-related macular degeneration and the role of the complement system. Mol Immunol.

[80] Assady S, Wanner N, Skorecki KL, Huber TB (2017). New insights into podocyte biology in glomerular health and disease. J Am Soc Nephrol.

[81] Mundel P (2002). Podocyte biology and response to injury. J Am Soc Nephrol.

[82] Grahammer F (2017). New structural insights into podocyte biology. Cell Tissue Res.

[83] Zoja C, Buelli S, Morigi M (2019). Shiga toxin triggers endothelial and podocyte injury: the role of complement activation. Pediatr Nephrol.

[84] dos Santos M, Poletti PT, Milhoransa P, Monticielo OA, Veronese FV (2017). Unraveling the podocyte injury in lupus nephritis: clinical and experimental approaches. Semin Arthritis Rheum.

[85] Sakhi H, Moktefi A, Bouachi K, Audard V, Hénique C, Remy P (2019). Podocyte injury in lupus nephritis. J Clin Med.

[86] Sharma M, Vignesh P, Tiewsoh K, Rawat A (2020). Revisiting the complement system in systemic lupus erythematosus. Expert Rev Clin Immunol.

[87] Bao L, Haas M, Quigg RJ (2011). Complement factor H deficiency accelerates development of lupus nephritis. J Am Soc Nephrol.

[88] Ronco P, Debiec H (2020). Molecular pathogenesis of membranous nephropathy. Annu Rev Pathol.

[89] Ronco P, Plaisier E, Debiec H (2021) Advances in membranous nephropathy. J Clin Med 10(4):607 10.3390/jcm10040607 PMC7915386 33562791

[90] Cattran DC, Brenchley PE (2017). Membranous nephropathy: integrating basic science into improved clinical management. Kidney Int.

[91] Qi Y-Y, Zhou X-J, Cheng F-J, Hou P, Ren Y-L, Wang S-X (2018). Increased autophagy is cytoprotective against podocyte injury induced by antibody and interferon-α in lupus nephritis. Ann Rheum Dis.

